# Understanding the Real State of Human Adult Hippocampal Neurogenesis From Studies of Rodents and Non-human Primates

**DOI:** 10.3389/fnins.2020.00839

**Published:** 2020-08-11

**Authors:** Tatsunori Seki

**Affiliations:** Department of Histology and Neuroanatomy, Tokyo Medical University, Tokyo, Japan

**Keywords:** adult neurogenesis, human, hippocampus, rodent, comparative

## Abstract

The concept of adult hippocampal neurogenesis (AHN) has been widely accepted, and a large number of studies have been performed in rodents using modern experimental techniques, which have clarified the nature and developmental processes of adult neural stem/progenitor cells, the functions of AHN, such as memory and learning, and its association with neural diseases. However, a fundamental problem is that it remains unclear as to what extent AHN actually occurs in humans. The answer to this is indispensable when physiological and pathological functions of human AHN are deduced from studies of rodent AHN, but there are controversial data on the extent of human AHN. In this review, studies on AHN performed in rodents and humans will be briefly reviewed, followed by a discussion of the studies in non-human primates. Then, how data of rodent and non-human primate AHN should be applied for understanding human AHN will be discussed.

## Introduction

Adult hippocampal neurogenesis (AHN) is now widely studied in the neuroscience field, because newly born neurons induce large-scale neuronal circuit alterations that are reported to be involved in learning and memory (Lledo et al., 2006; Treves et al., 2008; Kempermann, 2011; Drew et al., 2013; Abrous and Wojtowicz, 2015), diseases, such as epilepsy, stroke, and mental disorders (Danzer, 2012; Eisch and Petrik, 2012; Bowers and Jessberger, 2016; Toda et al., 2019), and the regeneration of brain tissue (Sawada and Sawamoto, 2013; Jessberger, 2016; Peng and Bonaguidi, 2018). Such research progress has been achieved mostly in rodents using modern experimental techniques, but has not been accomplished in humans, because the approaches that can be applied to human research are limited.

Adult hippocampal neurogenesis in rodents was discovered in the 1960s by Joseph Altman (Altman, 1963; Altman and Das, 1965), but this revolutionary concept had not been fully accepted until the late 1990s (Gross, 2000; Altman, 2011; Kempermann, 2011; Seki, 2011). AHN was rediscovered in the early and mid 1990s by the development of new techniques, such as the labeling of newly generated cells by bromodeoxyuridine (BrdU), and immunohistochemistry using an antibody for polysialylated neural cell adhesion molecule (PSA-NCAM) (Seki and Arai, 1993b, 1995; Kuhn et al., 1996).

For demonstrating the existence of human AHN, the BrdU-labeling technique clearly played an important role. Eriksson et al. (1998) investigated postmortem hippocampi from cancer patients who received a BrdU infusion for diagnostic purposes, and demonstrated the presence of newly born neuron that are BrdU-labeled cells costained with the neuronal marker NeuN, indicating that neurons are born in the adult human hippocampus.

However, subsequent studies using BrdU in humans have not been performed to date, probably because of the toxicity of BrdU. Instead, many immunohistochemical studies using markers of stem/progenitor cells and immature neuronal markers (INMs) have been performed on the human hippocampus without BrdU labeling, and a large amount of data on human AHN has been accumulated (Gould, 2007; Yuan et al., 2014; Duque and Spector, 2019).

In recent years, an important question was raised as to the extent of human AHN, because an immunohistochemical study suggested a sharp decline in neurogenesis during the early postnatal period, and an undetectable level of neurogenesis in the adult human hippocampus (Sorrells et al., 2018), whereas other reports suggest persistent AHN in humans (Boldrini et al., 2018; Moreno-Jiménez et al., 2019; Tobin et al., 2019). These studies resulted in many controversial debates (Kempermann et al., 2018; Kuhn et al., 2018; Paredes et al., 2018; Tartt et al., 2018; Oppenheim, 2019; Snyder, 2019), because solving this problem is inevitable when physiological and pathological functions of human AHN are inferred on the basis of data on rodent AHN.

In this article, I will review the studies on AHN in rodents and humans, followed by those in non-human primates to fill the gap between rodents and humans. Furthermore, interpretation of the expression of INMs in these specimens to understand human AHN will also be discussed.

## AHN in Rodents

During the previous three decades, numerous studies on AHN have been performed in mice and rats. Particularly in mice, modern gene manipulation techniques demonstrated many aspects of AHN, such as the properties of stem/progenitor cells and their developmental processes and regulatory mechanisms, as well as their function in diseases associated with AHN (Gonçalves et al., 2016b; Semerci and Maletic-Savatic, 2016; Hochgerner et al., 2018; Toda et al., 2019). Therefore, most general knowledge of AHN comes from studies performed in mice.

### Properties of Adult Neural Stem Cells

To date, many studies on rodents have repeatedly indicated that granule cells are generated in the subgranular zone (SGZ) of the adult hippocampal dentate gyrus (DG), which is the narrow band of cells between the granule cell layer (GCL) and the hilus. The SGZ harbors adult neural stem cells (aNSCs) that express the stem cell markers nestin and Sox2, and demonstrate a radial morphology (Kriegstein and Alvarez-Buylla, 2009; Gebara et al., 2016). Furthermore, aNSCs have astrocytic features, including the expression of astrocyte markers, such as glial fibrillary acidic protein, brain lipid-binding protein, and glutamate-aspartate transporter (Seri et al., 2001), but not S100β expression (Seri et al., 2004; Steiner et al., 2004). However, it should be noted that although aNSCs express these molecular markers, not all cells positive for these markers are aNSCs, because astrocytes and non-aNSCs also express these markers (Von Bohlen Und Halbach, 2011; Zhang and Jiao, 2015).

### Neuronal Differentiation of Neural Stem Cells

Adult neural stem cells give rise to neurons via the proliferation of intermediate progenitors or the transient amplification of progenitor cells that can proliferate to self-renew a few times to produce neurons. It has been reported that early proliferating intermediate progenitor cells express Ascl1, Prox1, and Hu, and that late proliferating intermediate progenitor cells express PSA-NCAM, doublecortin (DCX), and NeuroD (Seki et al., 2007; Von Bohlen Und Halbach, 2011; Kempermann et al., 2015; Zhang and Jiao, 2015).

The morphology of neural progenitor cells differs from that of mature granule cells. Neural stem/progenitor cells proliferate and form clusters that contain from a few to more than 10 cells (Seki et al., 2007). The neuronal progenitor cells within the clusters are round or ovoid cells with short processes that are smaller than mature granule cells. These neural precursor cells migrate horizontally in the SGZ, to become horizontally oriented fusiform cells extending long horizontal processes (Seki et al., 2007; Pilz et al., 2018). They then settle in their final position, extending their thin apical dendrites.

### Maturation of Neuronal Precursor Cells

During the maturation process, newly generated neurons expressing INMs develop axons and branched dendrites, form synapses on them, and finally become mature granule cells (Hastings and Gould, 1999; Seki and Arai, 1999a, b; Brown et al., 2003; Faulkner et al., 2008; Sultan et al., 2015; Gonçalves et al., 2016a). Developing granule cells initially strongly express PSA-NCAM, and have few synaptic contacts on the cell surface of developing dendrites (Seki and Arai, 1999b) and axon terminals (Seki and Arai, 1999a). The expression of PSA-NCAM and DCX disappears from half of the newly generated cells by 4–6 weeks after their birth (Seki, 2002; Brown et al., 2003), and the developing granule cells become mature granule cells that have synaptic contacts. Because PSA-NCAM is known to prevent the formation of cell-cell contacts (Rutishauser, 2008), PSA-NCAM may interfere with synapse formation during dendrite development. The newly generated and developing granule cells are reported to have high excitability (Doetsch and Hen, 2005; Marin-Burgin et al., 2012), but the excitability decreases by 8 weeks after generation (Mongiat et al., 2009).

### Aging and AHN

Many reports demonstrate that the level of rodent AHN declines with age (Seki and Arai, 1995; Amrein et al., 2004; Ben Abdallah et al., 2010; Lee et al., 2012). The number of proliferating (Ki67+, PCNA+) cells, INM+ (DCX+, PSA-NCAM+) cells, and BrdU-labeled cells positive for neuronal markers are exponentially decreased during 2–6 months of age in rodents. However, thereafter, a small number of these cells persist during aging, and can be detected even in aged rodents, suggesting that rodent AHN occurs throughout life, but the level of AHN is low in middle-aged rodents.

## AHN in Humans

### Early Studies in the 1990s

The first suggestion of postnatal human neurogenesis was from a study in 1994, in which immunohistochemical staining was performed for PSA-NCAM in the structurally non-atrophic brains of children with extrahippocampal seizures. The study showed that in children with severe epilepsy, numerous PSA-NCAM+ immature neurons exist in the SGZ and GCL of the hippocampus of children younger than 2 years of age, but such neurons decrease in number by 6–8 years of age, and are undetectable in older children (>8 years of age) (Mathern et al., 1994, 2002).

However, another study in 1998 that performed PSA-NCAM immunohistochemistry on surgically removed hippocampi and entorhinal cortices of patients with drug-refractory temporal lobe epilepsy (mean age, 34 years) and autopsy controls (mean age, 47 years) demonstrated that a substantial number of PSA-NCAM+ cells are detectable in the SGZ of the adult human hippocampus, and that the number decreases in epileptic patients with severe neuronal damage (Mikkonen et al., 1998). Similarly, another study reported that PSA-NCAM+ cells were found in patients with Alzheimer disease (mean age, 82 years) and control patients (mean age, 71 years) (Mikkonen et al., 1999).

A study of postmortem human brains at the age of 7 months to 82 years with no obvious neuropathology demonstrated that strongly PSA-NCAM+ immature granule cells extending mossy fibers are found in the SGZ and GCL by 3 years of age (Ni Dhuill et al., 1999). After that, the number of PSA-NCAM+ cells decreased substantially, but numerous PSA-NCAM+ hilar neurons appeared at 2–3 years of age and persisted until the eighth decade of life. Although the exact reasons as to why the above three studies are inconsistent regarding the presence or amount of PSA-NCAM+ cells in adults remain unclear, it may be owing to differences in patients (with or without epilepsy), the severity of their seizures, sample conditions (postmortem brains or surgically removed hippocampi), and immunohistochemical methods.

A unique study of postmortem brains from adult cancer patients (average age: 64.4 ± 2.9 years) injected with BrdU for estimating the proliferation of tumor cells identified newly generated neurons in the adult human hippocampus, which had BrdU-labeled nuclei with NeuN+, calbindin+, and neuron specific enolase+ cell bodies (Eriksson et al., 1998). However, the extent of AHN, as reported in rodent quantitative fate-mapping experiments, remained unclear.

### Studies Between 2000 and 2017

As the use of BrdU is difficult in humans owing to its toxicity, for the detection of stem cells, proliferating cells, neural progenitors, and immature neurons in the human hippocampus, many researchers have performed immunohistochemistry for several molecular markers of proliferating cells, stem/progenitor cells, and immature neurons, which suggested the presence of AHN in the brains of healthy humans (Knoth et al., 2010; Dennis et al., 2016; Mathews et al., 2017) and patients with epilepsy (Liu et al., 2008), ischemia (Jin et al., 2006; Macas et al., 2006), Alzheimer disease (Jin et al., 2004; Liu and Song, 2016), and psychiatric diseases (Duque and Spector, 2019). Some studies have shown distinct age-dependent alterations in AHN.

An immunohistochemical study on postmortem human brains from birth to 100 years of age demonstrated that DCX+ cells are present in the SGZ and GCL during aging, but the number of DCX+ cells declines exponentially, and becomes very low by 2 years of age, and furthermore, that during aging, the appearance of DCX+ cells changes; i.e., at younger ages, DCX immunoreactivity is seen in apical dendrites, but at older ages, it is distributed only around the nucleus, suggesting that the expression pattern of DCX in the hippocampal neurogenic region is altered qualitatively and quantitatively with aging (Knoth et al., 2010).

Immunohistochemistry of Ki67 and DCX in the postmortem brains of subjects between the age of 0.2 and 59 years has shown that in infants, DCX+ cells are densely clustered in the GCL, but Ki67+ proliferating cells are distributed throughout the DG (Dennis et al., 2016). Thereafter, the number of clusters of DCX+ cells became very low by 3 years of age, and only a sparse distribution of DCX+ cells was seen in older juveniles and adults. Ki67+ proliferating cells were rarely seen in the SGZ. In addition, it has been reported that the mRNA levels of Ki67 and DCX in the healthy human hippocampus decreases throughout the lifespan of a human (Mathews et al., 2017). These data suggest that human AHN sharply decreases in infants.

### New Techniques in the 2000s

In addition to immunohistochemical techniques, nuclear magnetic resonance spectroscopy is used for non-invasively detecting biomarkers that are enriched in neural stem cells and neural progenitor cells in the live human brain, and has the possibility of identifying and quantifying adult human neural stem cells and progenitor cells (Bhardwaj et al., 2006; Manganas et al., 2007; Castiglione et al., 2017).

Attention has also been paid to new alternative approaches to estimate the levels of neurogenesis in humans, by measuring the concentration of nuclear bomb test-derived ^14^C in genomic DNA. Analysis using this method suggested that in adult humans, 700 new neurons are added to the hippocampus every day, and a large subpopulation of hippocampal neurons is exchanged throughout life, suggesting that humans and mice have similar levels of AHN (Spalding et al., 2013). The ^14^C birth dating method also showed the existence of neurogenesis in the adult striatum, and its absence in the adult human neocortex (Bhardwaj et al., 2006).

### Recent Conflicting Reports in 2018 and 2019

A recent study of postmortem brains from human fetal and postnatal subjects, and surgically resected samples from epileptic patients showed that the number of Ki67+/Sox2+ proliferating progenitors and DCX+/PSA-NCAM+ young neurons in the DG decrease sharply during the first year of life, and that neurogenesis in adults is undetectable (Sorrells et al., 2018). This study caused considerable debate (Kempermann et al., 2018; Kuhn et al., 2018; Paredes et al., 2018; Tartt et al., 2018; Snyder, 2019).

Soon after the above study, a study on hippocampi collected on autopsy demonstrated the persistence of proliferating neuronal progenitors and immature neurons, despite a decrease in the number of quiescent stem cells (Boldrini et al., 2018). An immunohistochemical study using improved tissue processing methods showed that AHN occurs frequently (Moreno-Jiménez et al., 2019). These improved immunohistochemical techniques revealed a substantial number of DCX+ cells in the human DG. A subpopulation of DCX+ cells were positive for PH3, Prox1, PSA-NCAM, calbindin, and calretinin, but they were often found in the upper and middle parts of the GCL in addition to the SGZ, which appears to resemble granule cells undergoing dematuration, as reported previously (Hagihara et al., 2019; Ohira et al., 2019). Persistent neurogenesis, which is shown by the presence of Nestin+/Sox2+/Ki67+ neural progenitors, DCX+/PCNA+ neuroblasts, and DCX+ immature neurons, was also reported in older adults and to a lesser extent, in Alzheimer disease patients (Tobin et al., 2019).

A part of the discrepancy in immunohistochemical studies appears to be caused by differences in techniques and specimens (Moreno-Jiménez et al., 2019). Most specimens were derived from postmortem brains with different postmortem intervals until fixation, as well as different methods of surgical removal of tissue. Regarding immunohistochemistry, there are differences in the methods of tissue preparation among the samples (paraffin or cryostat sections), and immunohistochemical procedures varied among the studies (with/without antigen retrieval pretreatment).

In this regard, a recent study using specimens surgically removed and immediately fixed, and subjected to antigen retrieval treatments clearly showed that a substantial number of PSA-NCAM+ neurons are distributed densely below the GCL, but the number of proliferating progenitors (Ki67+/HuB+/DCX+ cells) were very low (Seki et al., 2019). This suggests that immature-type neurons are not recently generated neurons, and the level of hippocampal neuronal production in adult humans is low. This conclusion raises the question as to the identity of INM+ cells, which will be discussed later.

## AHN in Non-Human Primates

Knowledge regarding AHN in non-human primates is expected to bridge the gap between humans and rodents (Workman et al., 2013; Charvet and Finlay, 2018), because (1) analysis of exact cell division, and fate tracing using BrdU can be performed, (2) non-human primates are evolutionally the most close relatives of humans, and (3) some non-human primate species have a much longer lifespan than rodents. The following information of the ages of their first reproduction and the lifespan of non-human primates was obtained from previous studies (Gould et al., 1997; Amrein et al., 2011).

In the late 1990s, the occurrence of AHN in non-human primates was demonstrated in some types of monkeys using BrdU labeling and markers for proliferating cells (PCNA), mature neurons (neuron-specific enolase, NeuN), and immature neurons (TuJ1, TOAD-64). The first indication of AHN in an animal close to primates was from treeshrews (*Tupaia belangeri*) at the age of 7 months to 2.5 years (age at first reproduction = 4–5 months, lifespan = up to 12 years in captivity) (Gould et al., 1997). After that, the presence of AHN in non-human primates was reported in common marmoset monkeys (*Callithrix jacchus*) at the age of 3 years (age at first reproduction = 595 days, average lifespan = 12 years) (Gould et al., 1998), and macaque monkeys (*Macaca mulatta* and *Macaca fasciculata*) at the age of 5.5–16.5 years (Kornack and Rakic, 1999) and at the age of 5–23 years (Gould et al., 1999), respectively (*M. mulatta*, age at first reproduction = 1,279 days, average lifespan = 14 years; *Macaca fascicularis*, age at first reproduction = 1,410 days, average lifespan = 14 years).

In the 2000s, several reports on non-human primates indicated that the AHN is affected by stress (Gould et al., 1998), antidepressants (Perera et al., 2007), and ischemia (Tonchev et al., 2003; Yamashima et al., 2004; Koketsu et al., 2006). Non-human primate AHN may also be involved in memory and learning (Aizawa et al., 2009), but AHN and learning ability are reported to be moderately associated with each other (Ngwenya et al., 2015).

### Absolute, but Not Relative, Age-Dependent Decrease in Neuronal Production

An early study in macaque monkeys showed that although neuronal production was found in both young and aged monkeys, the numbers of BrdU-labeled proliferating cells and TOAD-64-expressing immature neurons were much lower in aged monkeys than in young monkeys (Gould et al., 1999). Thereafter, detailed quantitative studies repeatedly confirmed the age-associated exponential decline in adult non-human primate neurogenesis in common marmosets (Leuner et al., 2007) and macaque monkeys (Aizawa et al., 2009, 2011; Amrein et al., 2011; Ngwenya et al., 2015).

Comparison of the age-dependent decrease in neurogenesis between rodents and non-human primates has demonstrated an important hypothetical concept that the decrease in neurogenesis, particularly proliferation of progenitor cells, is regulated by absolute age, but not by relative age (Kornack and Rakic, 1999; Amrein et al., 2011). There is a considerable difference in lifespan between rodents and non-human primates, i.e., the lifespan of mice and rats is 1.5–2.5 years, whereas the lifespan of rhesus monkeys (*M. mulatta*) is 14 years. Nevertheless, in both rodents and monkeys, the numbers of proliferating cells are exponentially reduced by 2–3 years of age, although at 2 years of age, rodents are aged animals, but rhesus monkeys are infants. Thus, a decrease in postnatal neurogenesis occurs in rodents at middle to older stages, but in macaque monkeys at infant stages.

### Prolonged Maturation of Newly Generated Neurons in Non-human Primates

The detailed developmental processes of the generation of new neurons in rhesus monkeys have been reported (Ngwenya et al., 2006, 2008, 2015; Kohler et al., 2011). An important phenomenon in non-human primates is the prolonged time required for the maturation of newly generated granule cells compared with rodents. The period required for maturation was estimated using the time when the expression of INMs, i.e., PSA-NCAM and DCX, disappears from BrdU-labeled newly generated cells.

In rodents, most new BrdU-labeled neurons lost PSA-NCAM and DCX immunoreactivity by 1–1.5 months after BrdU injection, indicating that the maturation of newly born granule cells occurs by 1–1.5 months (Seki, 2002; Brown et al., 2003; Kempermann et al., 2003). However, in the case of adult macaque monkeys, only half of the BrdU-labeled newly generated neurons lost their DCX expression by 6 months, and during this time period, new neurons continued to develop their dendrites, suggesting that maturation of new granule cells at the structural and molecular levels takes more than 6 months (Kohler et al., 2011; Ngwenya et al., 2015). This suggests that newly generated neurons in non-human primates have substantial plasticity during a long period. This prolonged immature state may compensate for the decrease in plasticity caused by a rapid decrease in neurogenesis in non-human primates.

Furthermore, a recent report has shown that an increase in DCX+ dentate granule cells without an increase in neuronal production is induced by fluoxetine treatment in the common marmoset, which suggests that mature granule cells are able to re-express INMs, which is a phenomenon called dematuration (Ohira et al., 2019).

## Validity of INMS as Proxy Markers for Adult Neurogenesis

Not all PSA-NCAM and DCX-expressing neurons are newly generated neurons in the adult brain. The existence of PSA-NCAM+ and DCX+ neurons in non-neurogenic regions of the adult brain has been reported in various mammals, including rodents, non-human primates, and humans (La Rosa et al., 2019). BrdU-labeling studies clearly demonstrated that the INM+ neurons in non-neurogenic regions are generated during the embryonic periods (Gómez-Climent et al., 2008; Luzzati et al., 2009; Rubio et al., 2016).

In adult mice, rats, guinea pigs, and rabbits, PSA-NCAM+ and DCX+ cells are found in the paleocortex (piriform and entorhinal cortices) (Seki and Arai, 1991; Bonfanti et al., 1992; Nacher et al., 2001; Gómez-Climent et al., 2008; Klempin et al., 2011), cingulate cortex (Gómez-Climent et al., 2011), association cortex (Luzzati et al., 2009), and spinal cord (Seki and Arai, 1993a). Additionally, it was also reported that even in the hippocampus, PSA-NCAM+ neurons are seen in non-neurogenic regions, such as the hilus, CA1/3, and subiculum (Nacher et al., 2002). These accumulated data indicate that PSA-NCAM+ and DCX+ cells in these non-neurogenic regions consist of a subpopulation of interneurons, and have immature characteristics in their structures, including less dendritic arborization, spine density, and synaptic contacts (Gómez-Climent et al., 2008, 2011; Guirado et al., 2014). A recent fate-mapping study using DCX-CreER^T2^/Flox-EGFP transgenic mice demonstrated that DCX+ neurons in the adult piriform cortex progressively resume the development of dendrites, axons, and synaptic contacts during aging (La Rosa et al., 2019).

INM+ cells in non-neurogenic regions are also observed in mammals with a large brain and long lifespan. In adult sheep, a study using INMs and BrdU labeling demonstrated that DCX+ cells are present in the external capsule and the surrounding gray matter (claustrum and amygdala), in addition to the piriform cortex and neocortex (Piumatti et al., 2018). In non-human primates, DCX+ neurons are found in non-neurogenic regions, such as the amygdala, entorhinal cortex, inferior temporal gyrus, and medial orbital gyrus (Zhang et al., 2009). DCX+ neurons generally coexpress PSA-NCAM, and some of them express neuron-specific nuclear protein and γ-aminobutyric acid, suggesting that they contain interneurons. Furthermore, similarly to INM+ cells in the neurogenic region, these cells demonstrate an age-dependent decrease in number. It has been proposed that mammals with a large brain and long lifespan have more INM+ non-newly generated neurons than mammals with a small brain and short lifespan (La Rosa et al., 2019).

In humans, DCX+ and PSA-NCAM+ cells have been identified in non-neurogenic regions of the neocortex (Varea et al., 2007; Srikandarajah et al., 2009), amygdala (Sorrells et al., 2019), and brain stem (Yoshimi et al., 2005). Furthermore, PSA-NCAM+ cells have been found in the non-neurogenic regions of the hippocampal formation, such as the CA1/3, subiculum, and entorhinal cortex (Mikkonen et al., 1998, 1999; Seki et al., 2019).

Although the exact nature and function of these INM+ cells in non-neurogenic regions remain unclear, some interesting hypotheses have been proposed, for example, the cells are in a state of arrested development (Gomez-Climent et al., 2010), in the process of continuous maturation of dormant precursors (Rotheneichner et al., 2018), and are a reservoir of young cells for the adult/aging brain (La Rosa et al., 2019).

Doublecortin and PSA-NCAM are generally used as proxy markers for AHN, but they should be considered as markers indicating immaturity or plasticity among both newly generated neurons and non-newly generated neurons. DCX, a microtubule-associated protein that is involved in the extension of neuronal processes, and PSA-NCAM, a cell-surface molecule that is implicated in cell movement and recognition of the cell surface, may be required for structural changes of both newly generated and non-newly generated neurons. Therefore, it should be noted that the level of neuronal production and neurogenesis cannot be evaluated solely by immunohistochemistry for INMs, and also that DCX, as well as PSA-NCAM, are not faultless molecular markers in terms of detecting newly or recently generated neurons (Verwer et al., 2007).

## The Real State of Human AHN

What do the studies on rodent and non-human primate AHN tell us about the real state of human AHN? These studies show the following: (1) proliferation of neural progenitor cells to produce new granule neurons in the SGZ of the hippocampus is decreased with absolute age, but not with relative age, which suggests that neuronal production in the human SGZ is also decreased with absolute age, as in non-human primates, (2) the maturation period of newly generated granule cells is much longer in primates than in rodents, which suggests that newly generated dentate granule cells in humans have a longer maturation period than those in rodents, and possibly than those in non-human primates, (3) INM+ cells in non-newly generated cells are detected in several adult brain regions of several mammalian species, which suggests the possible existence of similar immature-type neurons in the human hilus, SGZ, and GCL, in addition to INM+ adult-born or recently generated granule cells.

**FIGURE 1:**
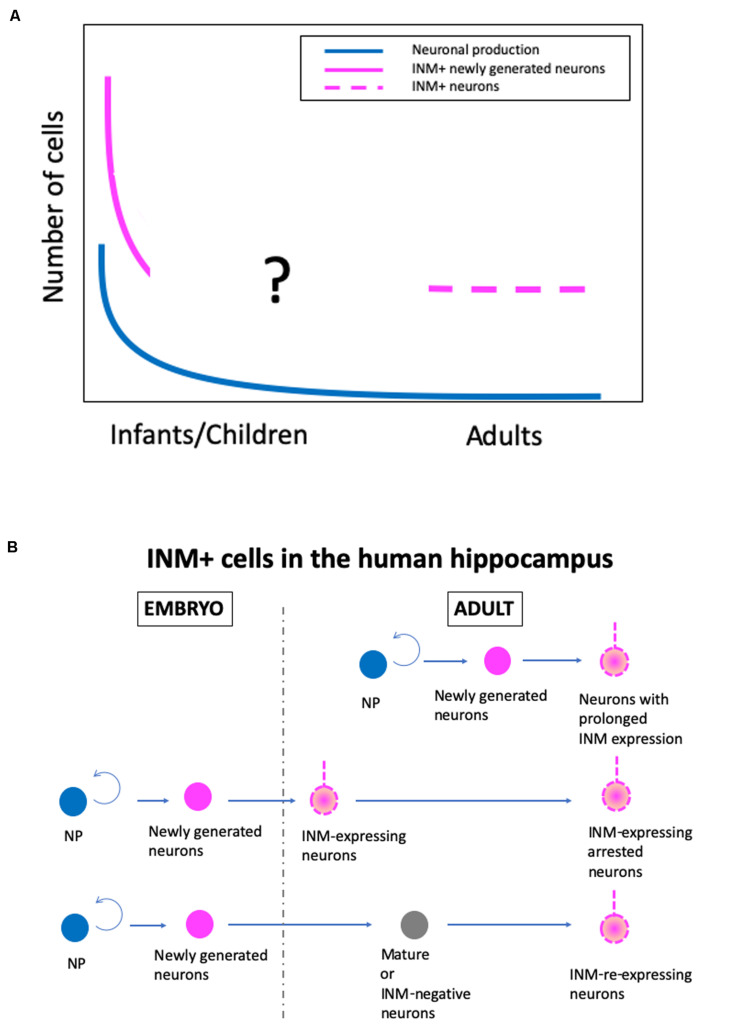
A new hypothetical model of the real state of human adult hippocampal neurogenesis. **(A)** Changes in the levels of neuronal production, immature neuronal marker-positive (INM+) newly generated neurons, and INM+ neurons in the adult human SGZ during aging. **(B)** The origin of INM+ neurons in the adult human hippocampus. NP, proliferating neural progenitor cells.

Although there are conflicting conclusions on human AHN, there is some agreement, as follows: (1) postnatal neuronal production in the SGZ occurs at a substantial level up to a few years after birth, but declines sharply, and (2) a substantial number of INM-expressing neurons exist in the adult human SGZ, although the detection of DCX+ cells depends on immunohistochemical methods, particularly antigen retrieval procedures (Figure 1A).

With the cumulative knowledge from rodents, non-human primates, and humans, as well as the data from our recent study in humans (Seki et al., 2019), I have developed a hypothesis about the real state of human AHN (Figure 1). In humans, there are at least two stages of postnatal hippocampal neurogenesis, i.e., the infant stage with a substantial level of neuronal production, and the adult stage with a very low level of neuronal production and a substantial number of INM-expressing cells, although the exact change in the number of INM+ cells during infant to adult stages is unknown. Additionally, regarding the origin of INM-expressing cells in the SGZ of the adult human hippocampus, there are three possible origins. The INM-expressing neurons are derived from (1) neurons with prolonged INM expression that are generated by adult neural progenitor cells and continue to express INMs over a prolonged period of time, (2) INM-expressing arrested neurons that are produced by embryonic neural progenitor cells and maintain immaturity, such as INM expression, and (3) INM-re-expressing neurons that are converted from mature or INM-negative adult neurons that are generated during the embryonic period (Figure 1B). Even though INM-expressing cells in the human adult hippocampus have different origins, they may have similar plasticity and compensate for the decline in plasticity by a low level of neurogenesis.

## Application of Rodent Studies to Understand Human “Postnatal” Hippocampal Neurogenesis

Next, I would like to propose some possible roles of “infant” and “adult” human hippocampal neurogenesis. In other words, there are possible ways to apply the vast accumulating knowledge about rodent AHN to understanding the functions of human AHN. Are learning and memory functions associated with postnatal neurogenesis in humans? An important point in postnatal neurogenesis is that new neurons are born and develop under circumstances in which various environmental sensory stimuli and locomotion information are inputted into hippocampal neuronal circuits (Lledo et al., 2006). This is very different from the condition of prenatal neurogenesis, which is not affected in principle by these inputs. In this sense, there is no difference between AHN in rodents and early postnatal neurogenesis in humans. Even though hippocampal neuronal production is limited to the early postnatal period in humans, studies on adult rodent neurogenesis are still worth performing to understand neurogenesis-associated learning and memory mechanisms in humans during the early postnatal period, when infants receive various sensory inputs every day and learn effectively from their experiences. Another possibility is that even if a very small number of neurons are newly generated in the adult human hippocampus, the accumulation of these new neurons, which have prolonged expression of INMs, must play important roles in memory and learning.

Do diseases affect postnatal neurogenesis in humans? Epilepsy is well known to be induced more frequently in children than in adults in humans (Holmes, 1997; Porter, 2008). Epileptic seizures should cause abnormal structural changes in newly generated neurons in infants and children, as in adult rodents, and subsequently must induce serious brain damage in the adult human hippocampus. Even if most PSA-NCAM+ and DCX+ cells in the adult human hippocampus are not recently generated neurons, these neurons that have higher plasticity than mature neurons can be easily altered structurally and physiologically by an epileptic state. In fact, in both epileptic patients and epileptic model rodents, the abnormal structure of PSA-NCAM+ neurons are very similar to each other (Seki et al., 2019). Furthermore, some diseases, such as Alzheimer disease (Tobin et al., 2019) and epilepsy (Seki et al., 2019), are reported to show a decrease in the number of INM+ neurons, regardless of whether the INM+ cells are recently generated neurons or non-recently generated neurons.

Taken together, studies on AHN are entering a new era, in which knowledge of rodent studies are not simply applied to understand human AHN, but species differences in brain size, lifespan, and ways of life, and identity of INM-expressing cells must be considered to understand the true state and function of human AHN (Amrein et al., 2011; Faykoo-Martinez et al., 2017; La Rosa et al., 2019; Oppenheim, 2019; Seki et al., 2019; Snyder, 2019). Furthermore, comprehensive analyses of postnatally born neurons, both in infants and in adults, and INM-expressing neurons, regardless of their origin, will enable us to understand the state and function of human AHN and plasticity.

## Author Contributions

The author confirms being the sole contributor of this work and has approved it for publication.

## Conflict of Interest

The author declares that the research was conducted in the absence of any commercial or financial relationships that could be construed as a potential conflict of interest.

## References

[B1] Abrous D. N. Wojtowicz J. M. (2015). Interaction between neurogenesis and hippocampal memory system: new vistas. *Cold Spring Harb. Perspect. Biol.* 7:a018952. 10.1101/cshperspect.a018952 26032718 PMC4448601

[B2] Aizawa K. Ageyama N. Terao K. Hisatsune T. (2011). Primate-specific alterations in neural stem/progenitor cells in the aged hippocampus. *Neurobiol. Aging* 32 140–150. 10.1016/j.neurobiolaging.2008.12.011 19201065

[B3] Aizawa K. Ageyama N. Yokoyama C. Hisatsune T. (2009). Age-dependent alteration in hippocampal neurogenesis correlates with learning performance of macaque monkeys. *Exp. Anim.* 58 403–407. 10.1538/expanim.58.403 19654438

[B4] Altman J. (1963). Autoradiographic investigation of cell proliferation in the brains of rats and cats. *Anat. Rec.* 145 573–591. 10.1002/ar.1091450409 14012334

[B5] Altman J. (2011). “The Discovery of Adult Mammalian Neurogenesis,” in *Neurogenesis in the Adult Brain*, eds Seki T. Sawamoto K. Parent J. M. Alvarez-Buylla A. (Tokyo: Springer), 3–46. 10.1007/978-4-431-53933-9_1

[B6] Altman J. Das G. D. (1965). Autoradiographic and histological evidence of postnatal hippocampal neurogenesis in rats. *J. Comp. Neurol.* 124 319–335. 10.1002/cne.901240303 5861717

[B7] Amrein I. Isler K. Lipp H. P. (2011). Comparing adult hippocampal neurogenesis in mammalian species and orders: influence of chronological age and life history stage. *Eur. J. Neurosci.* 34 978–987. 10.1111/j.1460-9568.2011.07804.x 21929629

[B8] Amrein I. Slomianka L. Poletaeva I. I. Bologova N. V. Lipp H. P. (2004). Marked species and age-dependent differences in cell proliferation and neurogenesis in the hippocampus of wild-living rodents. *Hippocampus* 14 1000–1010. 10.1002/hipo.20018 15390172

[B9] Ben Abdallah N. M. B. Slomianka L. Vyssotski A. L. Lipp H. P. (2010). Early age-related changes in adult hippocampal neurogenesis in C57 mice. *Neurobiol. Aging* 31 151–161. 10.1016/j.neurobiolaging.2008.03.002 18455269

[B10] Bhardwaj R. D. Curtis M. A. Spalding K. L. Buchholz B. A. Fink D. Björk-Eriksson T. (2006). Neocortical neurogenesis in humans is restricted to development. *Proc. Natl. Acad. Sci. U.S.A.* 103 12564–12568. 10.1073/pnas.0605177103 16901981 PMC1567918

[B11] Boldrini M. Fulmore C. A. Tartt A. N. Simeon L. R. Pavlova I. Poposka V. (2018). Human hippocampal neurogenesis persists throughout aging. *Cell Stem Cell* 22 589–599. 10.1016/j.stem.2018.03.015 29625071 PMC5957089

[B12] Bonfanti L. Olive S. Poulain D. A. Theodosis D. T. (1992). Mapping of the distribution of polysialylated neural cell adhesion molecule throughout the central nervous system of the adult rat: an immunohistochemical study. *Neuroscience* 49 419–436. 10.1016/0306-4522(92)90107-d 1436474

[B13] Bowers M. Jessberger S. (2016). Linking adult hippocampal neurogenesis with human physiology and disease. *Dev. Dyn.* 245 702–709. 10.1002/dvdy.24396 26890418

[B14] Brown J. P. Couillard-Despres S. Cooper-Kuhn C. M. Winkler J. Aigner L. Kuhn H. G. (2003). Transient expression of doublecortin during adult neurogenesis. *J. Comp. Neurol.* 467 1–10. 10.1002/cne.10874 14574675

[B15] Castiglione F. Ferro M. Mavroudakis E. Pellitteri R. Bossolasco P. Zaccheo D. (2017). NMR metabolomics for stem cell type discrimination. *Sci. Rep.* 7:15808. 10.1038/s41598-017-16043-8 29150616 PMC5693937

[B16] Charvet C. J. Finlay B. L. (2018). Comparing adult hippocampal neurogenesis across species: translating time to predict the tempo in humans. *Front. Neurosci.* 12:706. 10.3389/fnins.2018.00706 30344473 PMC6182078

[B17] Danzer S. C. (2012). Depression, stress, epilepsy and adult neurogenesis. *Exp Neurol* 233 22–32. 10.1016/j.expneurol.2011.05.023 21684275 PMC3199026

[B18] Dennis C. V. Suh L. S. Rodriguez M. L. Kril J. J. Sutherland G. T. (2016). Human adult neurogenesis across the ages: an immunohistochemical study. *Neuropathol. Appl. Neurobiol.* 42 621–638. 10.1111/nan.12337 27424496 PMC5125837

[B19] Doetsch F. Hen R. (2005). Young and excitable: the function of new neurons in the adult mammalian brain. *Curr. Opin. Neurobiol.* 15 121–128. 10.1016/j.conb.2005.01.018 15721754

[B20] Drew L. J. Fusi S. Hen R. (2013). Adult neurogenesis in the mammalian hippocampus: Why the dentate gyrus? *Learn. Mem.* 20 710–729. 10.1101/lm.026542.112 24255101 PMC3834622

[B21] Duque A. Spector R. (2019). A balanced evaluation of the evidence for adult neurogenesis in humans: implication for neuropsychiatric disorders. *Brain Struct. Funct.* 224 2281–2295. 10.1007/s00429-019-01917-6 31278571 PMC6852840

[B22] Eisch A. J. Petrik D. (2012). Depression and hippocampal neurogenesis: A road to remission? *Science* 338 72–75. 10.1126/science.1222941 23042885 PMC3756889

[B23] Eriksson P. S. Perfilieva E. Björk-Eriksson T. Alborn A. M. Nordborg C. Peterson D. A. (1998). Neurogenesis in the adult human hippocampus. *Nat. Med.* 4 1313–1317. 10.1038/3305 9809557

[B24] Faulkner R. L. Jang M. H. Liu X. B. Duan X. Sailor K. A. Kim J. Y. (2008). Development of hippocampal mossy fiber synaptic outputs by new neurons in the adult brain. *Proc. Natl. Acad. Sci. U.S.A.* 105 14157–14162. 10.1073/pnas.0806658105 18780780 PMC2544594

[B25] Faykoo-Martinez M. Toor I. Holmes M. M. (2017). Solving the neurogenesis puzzle: looking for pieces outside the traditional box. *Front. Neurosci.* 11:505. 10.3389/fnins.2017.00505 28943837 PMC5596094

[B26] Gebara E. Bonaguidi M. A. Beckervordersandforth R. Sultan S. Udry F. Gijs P. J. (2016). Heterogeneity of radial glia-like cells in the adult hippocampus. *Stem Cells* 34 997–1010. 10.1002/stem.2266 26729510 PMC5340291

[B27] Gomez-Climent M. A. Guirado R. Varea E. Nàcher J. (2010). “Arrested development”. Immature, but not recently generated, neurons in the adult brain. *Arch. Ital. Biol.* 148 159–172. 20830977

[B28] Gómez-Climent M. Á. Guirado R. Castillo-Gómez E. Varea E. Gutierrez-Mecinas M. Gilabert-Juan J. (2011). The polysialylated form of the neural cell adhesion molecule (PSA-NCAM) is expressed in a subpopulation of mature cortical interneurons characterized by reduced structural features and connectivity. *Cereb. Cortex* 21 1028–1041. 10.1093/cercor/bhq177 20843898

[B29] Gómez-Climent M. Á. A. Castillo-Gómez E. Varea E. Guirado R. Blasco-Ibáñez J. M. Crespo C. (2008). A population of prenatally generated cells in the rat paleocortex maintains an immature neuronal phenotype into adulthood. *Cereb. Cortex* 18 2229–2240. 10.1093/cercor/bhm255 18245040

[B30] Gonçalves J. T. Bloyd C. W. Shtrahman M. Johnston S. T. Schafer S. T. Parylak S. L. (2016a). In vivo imaging of dendritic pruning in dentate granule cells. *Nat. Neurosci.* 19 788–791. 10.1038/nn.4301 27135217 PMC4941946

[B31] Gonçalves J. T. Schafer S. T. Gage F. H. (2016b). Adult neurogenesis in the hippocampus: from stem cells to behavior. *Cell* 167 897–914. 10.1016/j.cell.2016.10.021 27814520

[B32] Gould E. (2007). How widespread is adult neurogenesis in mammals? *Nat. Rev. Neurosci.* 8 481–488. 10.1038/nrn2147 17514200

[B33] Gould E. McEwen B. S. Tanapat P. Galea L. A. Fuchs E. (1997). Neurogenesis in the dentate gyrus of the adult tree shrew is regulated by psychosocial stress and NMDA receptor activation. *J. Neurosci.* 17 2492–2498. 10.1523/jneurosci.17-07-02492.1997 9065509 PMC6573503

[B34] Gould E. Reeves A. J. Fallah M. Tanapat P. Gross C. G. Fuchs E. (1999). Hippocampal neurogenesis in adult Old World primates. *Proc. Natl. Acad. Sci. U.S.A.* 96 5263–5267. 10.1073/pnas.96.9.5263 10220454 PMC21852

[B35] Gould E. Tanapat P. McEwen B. S. Flugge G. Fuchs E. (1998). Proliferation of granule cell precursors in the dentate gyrus of adult monkey is diminished by stress. *Proc. Natil. Acad. Sci. U.S.A.* 95 3168–3171. 10.1073/pnas.95.6.3168 9501234 PMC19713

[B36] Gross C. G. (2000). Neurogenesis in the adult brain: death of a dogma. *Nat. Rev. Neurosci.* 1 67–73. 10.1038/35036235 11252770

[B37] Guirado R. Perez-Rando M. Sanchez-Matarredona D. Castillo-Gomez E. Liberia T. Rovira-Esteban L. (2014). The dendritic spines of interneurons are dynamic structures influenced by PSA-NCAM expression. *Cereb. Cortex* 24 3014–3024. 10.1093/cercor/bht156 23780867

[B38] Hagihara H. Murano T. Ohira K. Miwa M. Nakamura K. Miyakawa T. (2019). Expression of progenitor cell/immature neuron markers does not present definitive evidence for adult neurogenesis. *Mol. Brain* 12:108. 10.1186/s13041-019-0522-8 31823803 PMC6902531

[B39] Hastings N. B. Gould E. (1999). Rapid extension of axons into the CA3 region by adult-generated granule cells. *J. Comp. Neurol.* 413 146–154. 10.1002/(sici)1096-9861(19991011)413:1<146::aid-cne10>3.0.co;2-b 10540363

[B40] Hochgerner H. Zeisel A. Lönnerberg P. Linnarsson S. (2018). Conserved properties of dentate gyrus neurogenesis across postnatal development revealed by single-cell RNA sequencing. *Nat. Neurosci.* 21 290–299. 10.1038/s41593-017-0056-2 29335606

[B41] Holmes G. L. (1997). Epilepsy in the developing brain: lessons from the laboratory and clinic. *Epilepsia* 38 12–30. 10.1111/j.1528-1157.1997.tb01074.x 9024181

[B42] Jessberger S. (2016). Neural repair in the adult brain. *F1000Res.* 5:F1000 Faculty Rev-169. 10.12688/f1000research.7459.1 26918167 PMC4755395

[B43] Jin K. Peel A. L. Mao X. O. Xie L. Cottrell B. A. Henshall D. C. (2004). Increased hippocampal neurogenesis in Alzheimer’s disease. *Proc. Natl. Acad. Sci. U.S.A.* 101 343–347. 10.1073/pnas.2634794100 14660786 PMC314187

[B44] Jin K. Wang X. Xie L. Mao X. O. Zhu W. Wang Y. (2006). Evidence for stroke-induced neurogenesis in the human brain. *Proc. Natl. Acad. Sci. U.S.A.* 103 13198–13202. 10.1073/pnas.0603512103 16924107 PMC1559776

[B45] Kempermann G. (2011). *Adult Neurogenesis2.* Oxford: Oxford University Press.

[B46] Kempermann G. Gage F. H. Aigner L. Song H. Curtis M. A. Thuret S. (2018). Human adult neurogenesis: evidence and remaining questions. *Cell Stem Cell* 23 25–30. 10.1016/j.stem.2018.04.004 29681514 PMC6035081

[B47] Kempermann G. Gast D. Kronenberg G. Yamaguchi M. Gage F. H. (2003). Early determination and long-term persistence of adult-generated new neurons in the hippocampus of mice. *Development* 130 391–399. 10.1242/dev.00203 12466205

[B48] Kempermann G. Song H. Gage F. (2015). Neurogenesis in the adult hippocampus. *Cold Spring Harb. Perspect. Biol.* 7:a018812. 10.1101/cshperspect.a018812 26330519 PMC4563705

[B49] Klempin F. Kronenberg G. Cheung G. Kettenmann H. Kempermann G. (2011). Properties of doublecortin-(DCX)-expressing cells in the piriform cortex compared to the neurogenic dentate gyrus of adult mice. *PLoS One* 6:e25760. 10.1371/journal.pone.0025760 22022443 PMC3192736

[B50] Knoth R. Singec I. Ditter M. Pantazis G. Capetian P. Meyer R. P. (2010). Murine features of neurogenesis in the human hippocampus across the lifespan from 0 to 100 years. *PLoS One* 5:e8809. 10.1371/journal.pone.0008809 20126454 PMC2813284

[B51] Kohler S. J. Williams N. I. Stanton G. B. Cameron J. L. Greenough W. T. (2011). Maturation time of new granule cells in the dentate gyrus of adult macaque monkeys exceeds six months. *Proc. Natl. Acad. Sci. U.S.A.* 108 10326–10331. 10.1073/pnas.1017099108 21646517 PMC3121825

[B52] Koketsu D. Furuichi Y. Maeda M. Matsuoka N. Miyamoto Y. Hisatsune T. (2006). Increased number of new neurons in the olfactory bulb and hippocampus of adult non-human primates after focal ischemia. *Exp. Neurol.* 199 92–102. 10.1016/j.expneurol.2006.03.012 16712840

[B53] Kornack D. R. Rakic P. (1999). Continuation of neurogenesis in the hippocampus of the adult macaque monkey. *Proc. Natl. Acad. Sci. U.S.A.* 96 5768–5773. 10.1073/pnas.96.10.5768 10318959 PMC21935

[B54] Kriegstein A. Alvarez-Buylla A. (2009). The glial nature of embryonic and adult neural stem cells. *Annu. Rev. Neurosci.* 32 149–184. 10.1146/annurev.neuro.051508.135600 19555289 PMC3086722

[B55] Kuhn H. G. Dickinson-Anson H. Gage F. H. (1996). Neurogenesis in the dentate gyrus of the adult rat: age-related decrease of neuronal progenitor proliferation. *J. Neurosci.* 16 2027–2033. 10.1523/jneurosci.16-06-02027.1996 8604047 PMC6578509

[B56] Kuhn H. G. Toda T. Gage F. H. (2018). Adult hippocampal neurogenesis: a coming-of-age story. *J. Neurosci.* 38 10401–10410. 10.1523/JNEUROSCI.2144-18.2018 30381404 PMC6284110

[B57] La Rosa C. Ghibaudi M. Bonfanti L. (2019). Newly generated and non-newly generated “immature” neurons in the mammalian brain: a possible reservoir of young cells to prevent brain aging and disease? *J. Clin. Med.* 8:685. 10.3390/jcm8050685 31096632 PMC6571946

[B58] Lee S. W. Clemenson G. D. Gage F. H. (2012). New neurons in an aged brain. *Behav. Brain Res.* 227 497–507. 10.1016/j.bbr.2011.10.009 22024433 PMC3264739

[B59] Leuner B. Kozorovitskiy Y. Gross C. G. Gould E. (2007). Diminished adult neurogenesis in the marmoset brain precedes old age. *Proc. Natl. Acad. Sci. U.S.A.* 104 17169–17173. 10.1073/pnas.0708228104 17940008 PMC2040400

[B60] Liu H. Song N. (2016). Molecular mechanism of adult neurogenesis and its association with human brain diseases. *J. Cent. Nerv. Syst. Dis.* 8 5–11. 10.4137/JCNSD.S32204 27375363 PMC4915785

[B61] Liu Y. W. Curtis M. A. Gibbons H. M. Mee E. W. Bergin P. S. Teoh H. H. (2008). Doublecortin expression in the normal and epileptic adult human brain. *Eur. J. Neurosci.* 28 2254–2265. 10.1111/j.1460-9568.2008.06518.x 19046368

[B62] Lledo P. M. Alonso M. M. G. Grubb M. S. (2006). Adult neurogenesis and functional plasticity in neuronal circuits. *Nat. Rev. Neurosci.* 7 179–193. 10.1038/nrn1867 16495940

[B63] Luzzati F. Bonfanti L. Fasolo A. Peretto P. (2009). DCX and PSA-NCAM expression identifies a population of neurons preferentially distributed in associative areas of different pallial derivatives and vertebrate species. *Cereb. Cortex* 19 1028–1041. 10.1093/cercor/bhn145 18832334

[B64] Macas J. Nern C. Plate K. H. Momma S. (2006). Increased generation of neuronal progenitors after ischemic injury in the aged adult human forebrain. *J. Neurosci.* 26 13114–13119. 10.1523/JNEUROSCI.4667-06.2006 17167100 PMC6674966

[B65] Manganas L. Zhang X. Li Y. Hazel R. Smith S. Wagshul M. E. (2007). Magnetic resonance spectroscopy identifies neural progenitor cells in the live human brain. *Science* 318 980–985. 10.1126/science.1147851 17991865 PMC4039561

[B66] Marin-Burgin A. Mongiat L. A. Pardi M. B. Schinder A. F. (2012). Unique processing during a period of high excitation/inhibition balance in adult-born neurons. *Science* 335 1238–1242. 10.1126/science.1214956 22282476 PMC3385415

[B67] Mathern G. W. Leite P. J. Pretorius J. K. Quinn B. Peacock W. J. Babb T. L. (1994). Children with severe epilepsy: evidence of hippocampal neuron losses and aberrant mossy fiber sprouting during postnatal granule cell migration and differentiation. *Dev. Brain Res.* 78 70–80. 10.1016/0165-3806(94)90011-6 8004775

[B68] Mathern G. W. W. Leiphart J. L. L. De Vera A. Adelson P. D. D. Seki T. Neder L. (2002). Seizures decrease postnatal neurogenesis and granule cell development in the human fascia dentata. *Epilepsia* 43 68–73. 10.1046/j.1528-1157.2002.21601.x 12121298

[B69] Mathews K. J. Allen K. M. Boerrigter D. Ball H. Shannon Weickert C. Double K. L. (2017). Evidence for reduced neurogenesis in the aging human hippocampus despite stable stem cell markers. *Aging Cell* 16 1195–1199. 10.1111/acel.12641 28766905 PMC5595679

[B70] Mikkonen M. Soininen H. Kalvianen R. Tapiola T. Ylinen A. Vapalahti M. (1998). Remodeling of neuronal circuitries in human temporal lobe epilepsy: increased expression of highly polysialylated neural cell adhesion molecule in the hippocampus and the entorhinal cortex. *Ann. Neurol.* 44 923–934. 10.1002/ana.410440611 9851437

[B71] Mikkonen M. Soininen H. Tapiola T. Alafuzoff I. Miettinen R. (1999). Hippocampal plasticity in Alzheimer’s disease: changes in highly polysialylated NCAM immunoreactivity in the hippocampal formation. *Eur. J. Neurosci.* 11 1754–1764. 10.1046/j.1460-9568.1999.00593.x 10215928

[B72] Mongiat L. A. Espósito M. S. Lombardi G. Schinder A. F. (2009). Reliable activation of immature neurons in the adult hippocampus. *PLoS One* 4:e5320. 10.1371/journal.pone.0005320 19399173 PMC2670498

[B73] Moreno-Jiménez E. P. Flor-García M. Terreros-Roncal J. Rábano A. Cafini F. Pallas-Bazarra N. (2019). Adult hippocampal neurogenesis is abundant in neurologically healthy subjects and drops sharply in patients with Alzheimer’s disease. *Nat. Med.* 25 554–560. 10.1038/s41591-019-0375-9 30911133

[B74] Nacher J. Blasco-Ibanez J. M. McEwen B. S. (2002). Non-granule PSA-NCAM immunoreactive neurons in the rat hippocampus. *Brain Res.* 930 1–11. 10.1016/s0006-8993(01)03365-0 11879789

[B75] Nacher J. Crespo C. McEwen B. S. (2001). Doublecortin expression in the adult rat telencephalon. *Eur. J. Neurosci.* 14 629–644. 10.1046/j.0953-816x.2001.01683.x 11556888

[B76] Ngwenya L. B. Heyworth N. C. Shwe Y. Moore T. L. Rosene D. L. (2015). Age-related changes in dentate gyrus cell numbers, neurogenesis, and associations with cognitive impairments in the rhesus monkey. *Front. Syst. Neurosci.* 9:102. 10.3389/fnsys.2015.00102 26236203 PMC4500920

[B77] Ngwenya L. B. Peters A. Rosene D. L. (2006). Maturational sequence of newly generated neurons in the dentate gyrus of the young adult rhesus monkey. *J. Comp. Neurol.* 498 204–216. 10.1002/cne.21045 16856135

[B78] Ngwenya L. B. Rosene D. L. Peters A. (2008). An ultrastructural characterization of the newly generated cells in the adult monkey dentate gyrus. *Hippocampus* 18 210–220. 10.1002/hipo.20384 18058825

[B79] Ni Dhuill C. M. Fox G. B. Pittock S. J. O’Connell A. W. Murphy K. J. Regan C. M. (1999). Polysialylated neural cell adhesion molecule expression in the dentate gyrus of the human hippocampal formation from infancy to old age. *J. Neurosci. Res.* 55 99–106. 10.1002/(sici)1097-4547(19990101)55:1<99::aid-jnr11>3.0.co;2-s 9890438

[B80] Ohira K. Hagihara H. Miwa M. Nakamura K. Miyakawa T. (2019). Fluoxetine-induced dematuration of hippocampal neurons and adult cortical neurogenesis in the common marmoset. *Mol. Brain* 12:69. 10.1186/s13041-019-0489-5 31383032 PMC6683334

[B81] Oppenheim R. W. (2019). Adult hippocampal neurogenesis in mammals (and Humans): the death of a central dogma in neuroscience and its replacement by a new dogma. *Dev. Neurobiol.* 79 268–280. 10.1002/dneu.22674 30916471

[B82] Paredes M. F. Sorrells S. F. Cebrian-Silla A. Sandoval K. Qi D. Kelley K. W. (2018). Does adult neurogenesis persist in the human hippocampus? *Cell Stem Cell* 23 780–781. 10.1016/j.stem.2018.11.006 30526879 PMC6800191

[B83] Peng L. Bonaguidi M. A. (2018). Function and dysfunction of adult hippocampal neurogenesis in regeneration and disease. *Am. J. Pathol.* 188 23–28. 10.1016/j.ajpath.2017.09.004 29030053 PMC5745527

[B84] Perera T. D. Coplan J. D. Lisanby S. H. Lipira C. M. Arif M. Carpio C. (2007). Antidepressant-induced neurogenesis in the hippocampus of adult nonhuman primates. *J. Neurosci.* 27 4894–4901. 10.1523/JNEUROSCI.0237-07.2007 17475797 PMC6672102

[B85] Pilz G.-A. A. Bottes S. Betizeau M. Jörg D. J. Carta S. Simons B. D. (2018). Live imaging of neurogenesis in the adult mouse hippocampus. *Science* 359 658–662. 10.1126/science.aao5056 29439238 PMC6986926

[B86] Piumatti M. Palazzo O. La Rosa C. Crociara P. Parolisi R. Luzzati F. (2018). Non-Newly Generated, “Immature” neurons in the sheep brain are not restricted to cerebral cortex. *J. Neurosci.* 38 826–842. 10.1523/JNEUROSCI.1781-17.2017 29217680 PMC6596233

[B87] Porter B. E. (2008). Neurogenesis and epilepsy in the developing brain. *Epilepsia* 49 50–54. 10.1111/j.1528-1167.2008.01637.x 18522600 PMC2700768

[B88] Rotheneichner P. Belles M. Benedetti B. König R. Dannehl D. Kreutzer C. (2018). Cellular plasticity in the adult murine piriform cortex: continuous maturation of dormant precursors into excitatory neurons. *Cereb. Cortex* 28 2610–2621. 10.1093/cercor/bhy087 29688272 PMC5998952

[B89] Rubio A. Belles M. Belenguer G. Vidueira S. Fariñas I. Nacher J. (2016). Characterization and isolation of immature neurons of the adult mouse piriform cortex. *Dev. Neurobiol.* 76 748–763. 10.1002/dneu.22357 26487449

[B90] Rutishauser U. (2008). Polysialic acid in the plasticity of the developing and adult vertebrate nervous system. *Nat. Rev. Neurosci.* 9 26–35. 10.1038/nrn2285 18059411

[B91] Sawada M. Sawamoto K. (2013). Mechanisms of neurogenesis in the normal and injured adult brain. *Keio J. Med.* 62 13–28. 10.2302/kjm.2012-0005-RE 23563788

[B92] Seki T. (2002). Expression patterns of immature neuronal markers PSA-NCAM, CRMP-4 and NeuroD in the hippocampus of young adult and aged rodents. *J. Neurosci. Res.* 70 327–334. 10.1002/jnr.10387 12391592

[B93] Seki T. (2011). “From embryonic to adult neurogenesis in the dentate gyrus,” in *Neurogenesis in the Adult Brain*, eds Seki T. Sawamoto K. Parent J. M. Alvarez-Buylla A. (Tokyo: Springer), 193–216. 10.1007/978-4-431-53933-9_7

[B94] Seki T. Arai Y. (1991). Expression of highly polysialylated NCAM in the neocortex and piriform cortex of the developing and the adult rat. *Anat. Embryol.* 184 395–401. 10.1007/bf00957900 1952111

[B95] Seki T. Arai Y. (1993a). Highly polysialylated NCAM expression in the developing and adult rat spinal cord. *Brain Res. Dev. Brain Res.* 73 141–145. 10.1016/0165-3806(93)90056-g 7685664

[B96] Seki T. Arai Y. (1993b). Highly polysialylated neural cell adhesion molecule (NCAM-H) is expressed by newly generated granule cells in the dentate gyrus of the adult rat. *J. Neurosci.* 13 2351–2358. 10.1523/jneurosci.13-06-02351.1993 7684771 PMC6576495

[B97] Seki T. Arai Y. (1995). Age-related production of new granule cells in the adult dentate gyrus. *Neuroreport* 6 2479–2482. 10.1097/00001756-199512150-00010 8741746

[B98] Seki T. Arai Y. (1999a). Different polysialic acid-neural cell adhesion molecule expression patterns in distinct types of mossy fiber boutons in the adult hippocampus. *J. Comp. Neurol.* 410 115–125. 10.1002/(sici)1096-9861(19990719)410:1<115::aid-cne10>3.0.co;2-c 10397399

[B99] Seki T. Arai Y. (1999b). Temporal and spacial relationships between PSA-NCAM-expressing, newly generated granule cells, and radial glia-like cells in the adult dentate gyrus. *J. Comp. Neurol.* 410 503–513. 10.1002/(sici)1096-9861(19990802)410:3<503::aid-cne11>3.0.co;2-h 10404415

[B100] Seki T. Hori T. Miyata H. Maehara M. Namba T. (2019). Analysis of proliferating neuronal progenitors and immature neurons in the human hippocampus surgically removed from control and epileptic patients. *Sci. Rep.* 9:18194. 10.1038/s41598-019-54684-z 31796832 PMC6890740

[B101] Seki T. Namba T. Mochizuki H. Onodera M. (2007). Clustering, migration, and neurite formation of neural precursor cells in the adult rat hippocampus. *J. Comp. Neurol.* 502 275–290. 10.1002/cne 17348003

[B102] Semerci F. Maletic-Savatic M. (2016). Transgenic mouse models for studying adult neurogenesis. *Front. Biol.* 11 151–167. 10.1007/s11515-016-1405-3 28473846 PMC5412727

[B103] Seri B. García-Verdugo J. M. Collado-Morente L. McEwen B. S. Alvarez-Buylla A. (2004). Cell types, lineage, and architecture of the germinal zone in the adult dentate gyrus. *J. Comp. Neurol.* 478 359–378. 10.1002/cne.20288 15384070

[B104] Seri B. García-Verdugo J. M. McEwen B. S. Alvarez-Buylla A. (2001). Astrocytes give rise to new neurons in the adult mammalian hippocampus. *J. Neurosci.* 21 7153–7160. 10.1523/jneurosci.21-18-07153.2001 11549726 PMC6762987

[B105] Snyder J. S. (2019). Recalibrating the relevance of adult neurogenesis. *Trends Neurosci.* 42 164–178. 10.1016/j.tins.2018.12.001 30686490

[B106] Sorrells S. F. Paredes M. F. Cebrian-Silla A. Sandoval K. Qi D. Kelley K. W. (2018). Human hippocampal neurogenesis drops sharply in children to undetectable levels in adults. *Nature* 555 377–381. 10.1038/nature25975 29513649 PMC6179355

[B107] Sorrells S. F. Paredes M. F. Velmeshev D. Herranz-Pérez V. Sandoval K. Mayer S. (2019). Immature excitatory neurons develop during adolescence in the human amygdala. *Nat. Commun.* 10:2748. 10.1038/s41467-019-10765-1 31227709 PMC6588589

[B108] Spalding K. L. Bergmann O. Alkass K. Bernard S. Salehpour M. Huttner H. B. (2013). Dynamics of hippocampal neurogenesis in adult humans. *Cell* 153 1219–1227. 10.1016/j.cell.2013.05.002 23746839 PMC4394608

[B109] Srikandarajah N. Martinian L. Sisodiya S. M. Squier W. Blumcke I. Aronica E. (2009). Doublecortin expression in focal cortical dysplasia in epilepsy. *Epilepsia* 50 2619–2628. 10.1111/j.1528-1167.2009.02194.x 19583780

[B110] Steiner B. Kronenberg G. Jessberger S. Brandt M. D. Reuter K. Kempermann G. (2004). Differential regulation of gliogenesis in the context of adult hippocampal neurogenesis in mice. *Glia* 46 41–52. 10.1002/glia.10337 14999812

[B111] Sultan S. Li L. Moss J. Petrelli F. Cassé F. Gebara E. (2015). Synaptic integration of adult-born hippocampal neurons is locally controlled by astrocytes. *Neuron* 88 957–972. 10.1016/j.neuron.2015.10.037 26606999

[B112] Tartt A. N. Fulmore C. A. Liu Y. Rosoklija G. B. Dwork A. J. Arango V. (2018). Considerations for assessing the extent of hippocampal neurogenesis in the adult and aging human brain. *Cell Stem Cell* 23 782–783. 10.1016/j.stem.2018.10.025 30526880 PMC6830306

[B113] Tobin M. K. Musaraca K. Disouky A. Shetti A. Bheri A. Honer W. G. (2019). Human hippocampal neurogenesis persists in aged adults and Alzheimer’s disease patients. *Cell Stem Cell* 24 974. 10.1016/j.stem.2019.05.003 31130513 PMC6608595

[B114] Toda T. Parylak S. L. Linker S. B. Gage F. H. (2019). The role of adult hippocampal neurogenesis in brain health and disease. *Mol. Psychiatry* 24 67–87. 10.1038/s41380-018-0036-2 29679070 PMC6195869

[B115] Tonchev A. B. Yamashima T. Zhao L. Okano H. J. Okano H. (2003). Proliferation of neural and neuronal progenitors after global brain ischemia in young adult macaque monkeys. *Mol. Cell. Neurosci.* 23 292–301. 10.1016/s1044-7431(03)00058-7 12812760

[B116] Treves A. Tashiro A. Witter M. E. Moser E. I. (2008). What is the mammalian dentate gyrus good for? *Neuroscience* 154 1155–1172. 10.1016/j.neuroscience.2008.04.073 18554812

[B117] Varea E. Castillo-Gomez E. Gomez-Climent M. A. Blasco-Ibanez J. M. Crespo C. Martinez-Guijarro F. J. (2007). PSA-NCAM expression in the human prefrontal cortex. *J. Chem. Neuroanat.* 33 202–209. 10.1016/j.jchemneu.2007.03.006 17467233

[B118] Verwer R. W. H. Sluiter A. A. Balesar R. A. Baayen J. C. Noske D. P. Dirven C. M. F. (2007). Mature astrocytes in the adult human neocortex express the early neuronal marker doublecortin. *Brain* 130 3321–3335. 10.1093/brain/awm264 18055496

[B119] Von Bohlen Und Halbach O. (2011). Immunohistological markers for proliferative events, gliogenesis, and neurogenesis within the adult hippocampus. *Cell Tissue Res.* 345 1–19. 10.1007/s00441-011-1196-4 21647561

[B120] Workman A. D. Charvet C. J. Clancy B. Darlington R. B. Finlay B. L. (2013). Modeling transformations of neurodevelopmental sequences across mammalian species. *J. Neurosci.* 33 7368–7383. 10.1523/JNEUROSCI.5746-12.2013 23616543 PMC3928428

[B121] Yamashima T. Tonchev A. B. B. Vachkov I. H. H. Popivanova B. K. K. Seki T. Sawamoto K. (2004). Vascular adventitia generates neuronal progenitors in the monkey hippocampus after ischemia. *Hippocampus* 14 861–875. 10.1002/hipo.20001 15382256

[B122] Yoshimi K. Ren Y.-R. Y. R. Seki T. Yamada M. Ooizumi H. Onodera M. (2005). Possibility for neurogenesis in substantia nigra of parkinsonian brain. *Ann. Neurol.* 58 31–40. 10.1002/ana.20506 15912513

[B123] Yuan T. F. Li J. Ding F. Arias-Carrion O. (2014). Evidence of adult neurogenesis in non-human primates and human. *Cell Tissue Res.* 358 17–23. 10.1007/s00441-014-1980-z 25130142

[B124] Zhang J. Jiao J. (2015). Molecular biomarkers for embryonic and adult neural stem cell and neurogenesis. *Biomed Res. Int.* 2015:727542. 10.1155/2015/727542 26421301 PMC4569757

[B125] Zhang X.-M. Cai Y. Chu Y. Chen E.-Y. Feng J.-C. Luo X.-G. (2009). Doublecortin-expressing cells persist in the associative cerebral cortex and amygdala in aged nonhuman primates. *Front. Neuroanat.* 3:17. 10.3389/neuro.05.017.2009 19862344 PMC2766270

